# Evaluation of orthotic insoles for people with diabetes who are at-risk of first ulceration

**DOI:** 10.1186/s13047-019-0344-z

**Published:** 2019-06-18

**Authors:** Ana Martinez-Santos, Stephen Preece, Christopher J. Nester

**Affiliations:** University of Salford, Salford, UK

**Keywords:** Diabetic foot, Ulcer, Orthotic, CAD/CAM, Prevention

## Abstract

**Objective:**

This study focussed on pressure relieving orthotic insoles designed for retail footwear and people with diabetes and at risk of first forefoot ulceration. The aim was to investigate whether the pressure relieving effects of a customised metatarsal bar and forefoot cushioning are sensitive to bar location and shape, and material choice.

**Research design and methods:**

Patient-specific foot shape was used to design an orthotic insole, with metatarsal bar location and shape customised according to plantar pressure data. Changes in forefoot plantar pressure were investigated when 60 people with diabetes and neuropathy walked in nine variants of the orthotic insole. These comprised three variations in proximal/distal location of the customised metatarsal bar and three different metatarsal head offloading materials.

**Results & conclusions:**

The most frequent reductions in pressure occurred when the anterior edge of the metatarsal bar was placed at 77% of the peak pressure values, and its effects were independent of the choice of EVA or Poron offloading material. In the flat insole, 61% of participants had one or more metatarsal head areas with pressure above the 200 KPa, reducing to 58% when adopting generic orthotic design rules and 51% when using the best orthotic insole of the nine tested. Our results confirm that plantar pressure relief is sensitive to orthotic insole design decisions and individual patient feet.

## Background

Foot ulceration is estimated to affect 0.5–3% of the global population of people with diabetes [1] and the forefoot is the most commonly affected [2]. Given the seriousness of foot ulceration and that once established ulcers might only ever be in remission rather than cured [3], there is an increasing focus on preventing the first ulceration. Elevated plantar pressures are recognised as one of a range of risk factors for first ulceration and international guidelines advocate the use of footwear and orthotic insoles to reduce pressures [4, 5].

Suitably designed footwear is proven to reduce forefoot plantar pressures [6, 7] and risk of re-ulceration [8], but poor adherence is a key barrier to clinical success [9, 10]. Indeed, one trial observed a significant (19%) reduction in re-ulceration at 18-month follow up, but only in the subgroup with good adherence and who wore footwear as recommended [11]. Problems with adherence are likely to be more relevant for people without a history of ulceration because they may not consider themselves at risk [12]. They may, therefore, be less motivated to change their footwear from aesthetically pleasing retail shoes to pressure relieving designs incorporating stiff rocker soles or extra forefoot depth [12, 13]. For individuals at risk of ulceration but unwilling or unable to change their footwear, an orthotic insole used inside a retail shoe may still offer some protection against the risk of ulceration [14].

Suitably designed orthotic insoles have also been shown to reduce plantar pressures in patients at risk of plantar ulceration [15, 16]. Most studies have investigated the pressure relieving effects of elevations in areas of lower pressure (e.g. medial arch support and metatarsal bar [14–17] and use of soft materials in areas of high pressure (e.g. forefoot cushion) [18]. One difficulty is that studies typically test insoles which are too thick to be accommodated within retail footwear (e.g.10 mm [19], and 9 mm [20]) and thus may not be pertinent for prevention of first ulceration when retail footwear is likely the footwear of choice for patients.

A further difficulty is that preferred orthotic insole designs can rely on foot specific data that is difficult to collect in a routine clinical setting. Owings et al. [21] optimised pressure relief by using plantar pressure and foot shape data to inform metatarsal bar shape and location. However, few clinicians have access to pressure data and instead rely on manual techniques to estimate bar location and shape. Relatively small differences, or errors, in the location of offloading features (e.g. 5 mm), are thought to affect their efficacy [22–24]. Orthotic insole features that are effective but very sensitive variations between feet may demand a level of patient-specific customisation that is not achievable in routine practice.

A final issue is the implicit assumption in many studies that the lower the plantar pressure the better the footwear or insole design, whereas it might only be necessary to reduce pressure to below a safe threshold. Plantar pressure can be redistributed but not eradicated and reducing pressure at one location may simply displace risk of ulceration to a different area of the foot. In cases of re-ulceration, reducing plantar pressures to below 200 kPa has been advocated [25–27]. An equivalent threshold does not exist for first ulceration and 200 kPa may be too low given that pre-first ulceration plantar tissue is likely less vulnerable to external loads [28]. Knowing when lower pressures are ‘low enough’ is, therefore, a good strategy for footwear and insole evaluation.

This study focused on understanding the pressure relieving effects of orthotic insoles designed to fit inside retail footwear and targeted at people with diabetes and at risk of first forefoot ulceration. The aim was twofold. Firstly, to investigate whether the pressure relieving effect of a metatarsal bar and forefoot cushioning material is sensitive to bar location and material choice. We tested 9 different insole designs to investigate the separate and combined effects of bar location and material choice. With this data, we could propose group-optimised “best” bar location and material choice. We secondly sought to understand the extent to which this best but generic design choice met the < 200 kPa target compared to best performing insole design (out of the 9) for each individual participant. We would thus be able to comment on any added value of customising the bar location and material choice on a patient by patient basis.

## Methods

### Participants

Following ethical approval (REC: 13/NW/0331), 60 participants (40 male) were recruited via radio and health practices. The mean (SD) age was 67 [13] years and mean (SD) body mass index 29.41 (5.2) kg/m^2^. Inclusion criteria were aged ≥18, medically confirmed diagnosis of type 1 or 2 diabetes and signs of peripheral neuropathy.

The presence of neuropathy was assessed using a monofilament and a 128 Hz tuning fork whilst the participant had their eyes closed. The tuning fork was applied to internal and external malleoli and 1st and 5th metatarsal heads [29] and considered positive for neuropathy when one or more of the vibrations could not be sensed [30]. Light touch sensitivity was assessed using 10 g monofilaments tested in a random order on the 1st, 3rd and 5th toes, 1st, 3rd and 5th metatarsal heads, medial arch, lateral arch, heel and dorsum between 1st and 2nd toes. The test was positive for neuropathy if the patient could not feel the monofilament at one or more sites [31]. Exclusion criteria was a history of foot ulceration.

### Orthotic insole design

To design customised orthotic insoles 3D foot shape was collected using a scanner (Inescop, Spain) and plantar pressure data recorded using a platform (Emed® platform, Novel, Germany) while the subject stood. A set of nine customised orthotic insoles were designed using computer-aided design (iCAD PAN, Inescop, Spain). The 3D foot shape was used to customise the upper surface of a standardised 3D orthotic insole design (Salfordinsole Healthcare Ltd., UK). The orthotic insoles were 5 mm thick under the flat part of the forefoot area and metatarsal bars were an additional 5 mm above the flat area of the orthotic insole.

**Fig. 1:**
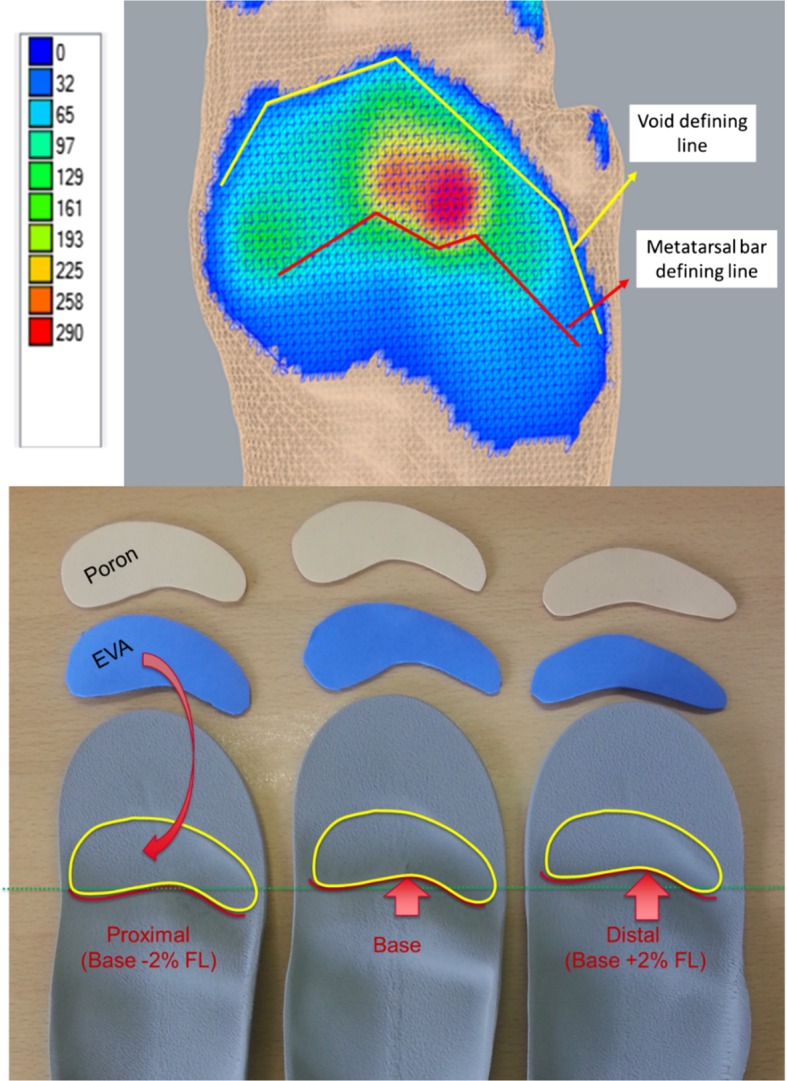
Design of the metatarsal bar and void and different orthotic insoles used for the study with different cushioning materials

The proximal/distal location of a metatarsal bar and a void (large cavity) distal to the bar was defined using the plantar pressure distribution. The location and shape of the distal edge of the metatarsal bar was defined by a line on the area where plantar pressure was 77% of the peak plantar pressure. This line also defined the proximal border of the void. The distal border of the void was distal to the area of peak plantar pressures and where pressure was < 10% of the peak plantar pressure (Fig. 1). The depth of the void was 3 mm.

Once the initial design of the metatarsal bar and void was completed, two variations on the design were created by moving the metatarsal bar proximal and distal by 2% of insole length. This percentage was chosen as it corresponds to a distance of 5 mm on a size 7 orthotic insole and was used in a previous study of metatarsal bars [32]. Orthotic insoles were made of medium density EVA (50° Shore A) and manufactured on a CNC milling machine.

Three different void conditions were created: EVA (20 Shore A), Poron (20 Shore A) and no material (i.e. empty void) and tailored pieces of EVA and Poron were prepared to fit the void for each participant. This created 9 different orthotic insole conditions: distal, middle and proximal metatarsal bar locations, each combined with EVA, Poron and no material variations for the void (Fig. 1).

### Data collection

**Fig. 2:**
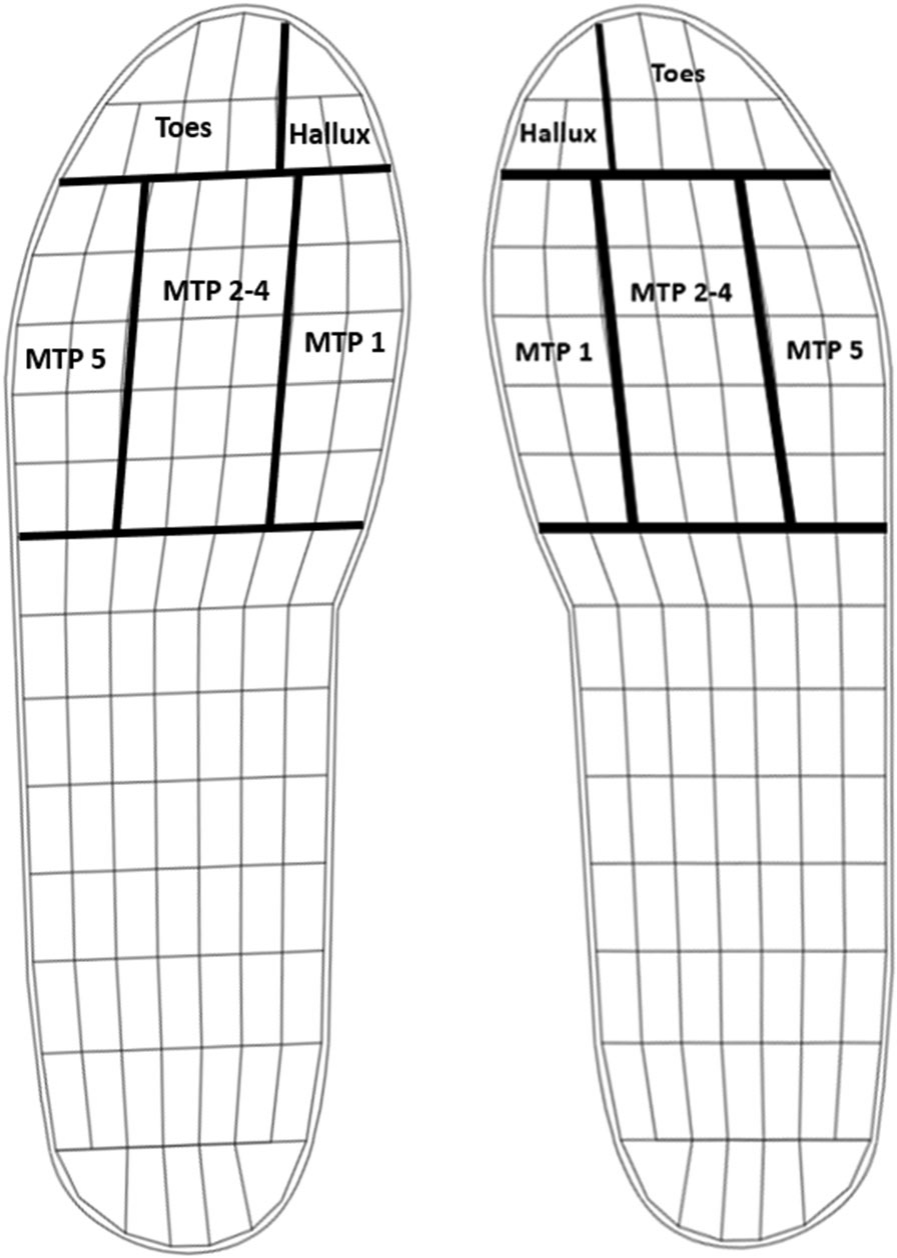
Pedar insoles sensor array and mask used for the pressure analysis

Novel Pedar-X system samplig at 50 Hz was used to collect in-shoe plantar pressure data. These insoles are composed of an array of 99 capacitive sensors arranged in rows and columns that enable monitoring the entire plantar area of the foot during walking (see Fig. 2). However, no reference to sensor size has been found in the scientific literature. While the system has been tested for accuracy and repeatability [33], the test protocol for regional pressure measurement did not utilize the sensors in the metatarsal region, which are key to this study. The absence of information regarding the dimensions of the Pedar sensors along with the uncertainty, albeit small, regarding accuracy of metatarsal pressure measurement with this system warrant further investigation in the future in order to meet optimal characteristics reported by Davis et al. [34].

Pressure data was collected whilst participants walked in each of the 9 orthotic insole designs plus a control insole (flat, 3 mm medium density EVA, 50° Shore A). Each insole was worn within a retail shoe with a removable insole. For men this was an Oxford style shoe and for females was a wedged style shoe. The pitch difference between the male and female shoes was 1.5 cm.

The order of testing was randomised and participants walked at a self-selected speed which was set prior to data collection using practice walks. Speed was monitored using optical timing gates and instructions offered to maintain walks within ±5% of the target speed. Participants completed a familiarisation period of between 2 and 5 min for each orthotic insole condition and a minimum of 20 steps were collected for each condition.

Peak plantar pressures were derived for the 1st metatarsophalangeal (MTP) joint, 2-4th metatarsal heads (MTH), the hallux, and 5th metatarsal head (see Fig. 2). The sensors corresponding to each region were defined according to Cavanagh and Ulbrecht [35] and peak pressures averaged across all steps to give a single pressure value for each foot region and orthotic insole condition for each participant. Statistical analysis showed common trends for both the left and right sides and therefore only data from the right side are presented.

### Statistical analysis

Statistical testing was conducted using SPSS (21.0). One-way repeated measures ANOVA testing was used to compare the peak pressures between the control insole and each of the nine contoured orthotic insoles. A two-way repeated measures ANOVA was conducted to compare the effect of varying metatarsal bar location and void material and identify any interaction between these two factors. If significant differences were found Bonferroni post hoc testing was used to explore pairwise differences using an alpha of 0.05. The number of participants with plantar pressures above 200 KPa was determined based on the area of highest pressure under the metatarsal heads, regardless of location.

## Results

In terms of pressure reductions compared to the control insole, the middle metatarsal bar location most frequently significantly reduced pressure at MTP 1 and MTP 2–4 and did so independent of the choice of EVA or Poron. The proximal bar location significantly reduced pressured when combined with Poron at both MTP 1 and MTP 2–4, and with EVA at MTP 2–4. The distal bar location significantly reduced pressure only when used with Poron and only at MTP 1. In terms of the material options, and compared to the control insole, only conditions involving EVA or Poron showed statistically significant reductions in pressure, and only at the 1st MTP and MTP 2–4. Hallux pressures were significantly elevated by all bar locations and all void conditions, whereas MTP 5 was not affected at all (Table 1).

**Table 1:** ANOVA statistics, in each anatomical region, for the main effects of material and metatarsal bar location and also for the interaction. Both the F-statistic and associated *p*-value have been reported. In addition, the 95% confidence intervals, and associated *p*-values, for the pairwise comparisons are included. Note that these p-values have been adjusted using a Bonferroni correction for multiple comparisons. All statistical differences (*p* < 0.05) have been marked with an*

	1st MTP	2–4 MTP	5th MTP	Hallux
Metatarsal bar location	F = 0.4, *p* = 0.655	F = 5.9, *p* < 0.003*	F = 0.9, *p* = 0.426	F = 3.2, *p* = 0.043*
Proximal vs middle	(−5.8, 12.8), *p* = 1.0	(−8.1, 10.9), *p* = 1.0	(−6.2, 2.7), *p* = 0.997	(−3.1, 11.4), *p* = 0.499
middle vs distal	(−16.4, 9.6), *p* = 1.0	(−27.2, 1.0), *p* = 0.031*	(−3.3, 8.3), *p* = 0.875	(−16.9, −0.2), *p* = 0.042*
Distal vs proximal	(−9.2, 9.1), *p* = 1.0	(2.4, 23), *p* = 0.011*	(−4.8, 3.4), *p* = 1.0	(−4.8, 13.6), *p* = 0.719
Material	F = 31.3, *p* < 0.001*	F = 41.4, *p* < 0.001*	F = 9.9, *p* < 0.001*	F = 0.03, *p* = 970
EVA vs Poron	(−2.09, 7.7), *p* = 0.48	(−4.2, 4.4), *p* = 1.0	(−3.3, 3.2), p = 1.0	(−5.6, 5.1), *p* = 1.0
Poron vs void	(−29.2, −11.9), *p* < 0.001*	(− 26.4, − 11.9), *p* < 0.001*	(−10.5, − 1.5), *p* = 0.005*	(− 5.5, 6.7), *p* = 1.0
Void vs EVA	(9.3, 23.2), *p* < 0.001*	(13.1, 24.9) *P* < 0.001*	(2.3, 9.9), *p* = 0.001*	(−6, 5.4), *p* = 1.0
Interaction	F = 0.4, *p* = 0.818	F = 0.5, *p* = 0.754	F = 1.4, *p* = 0.220	F = 0.6, *p* = 0.696

**Fig. 3:**
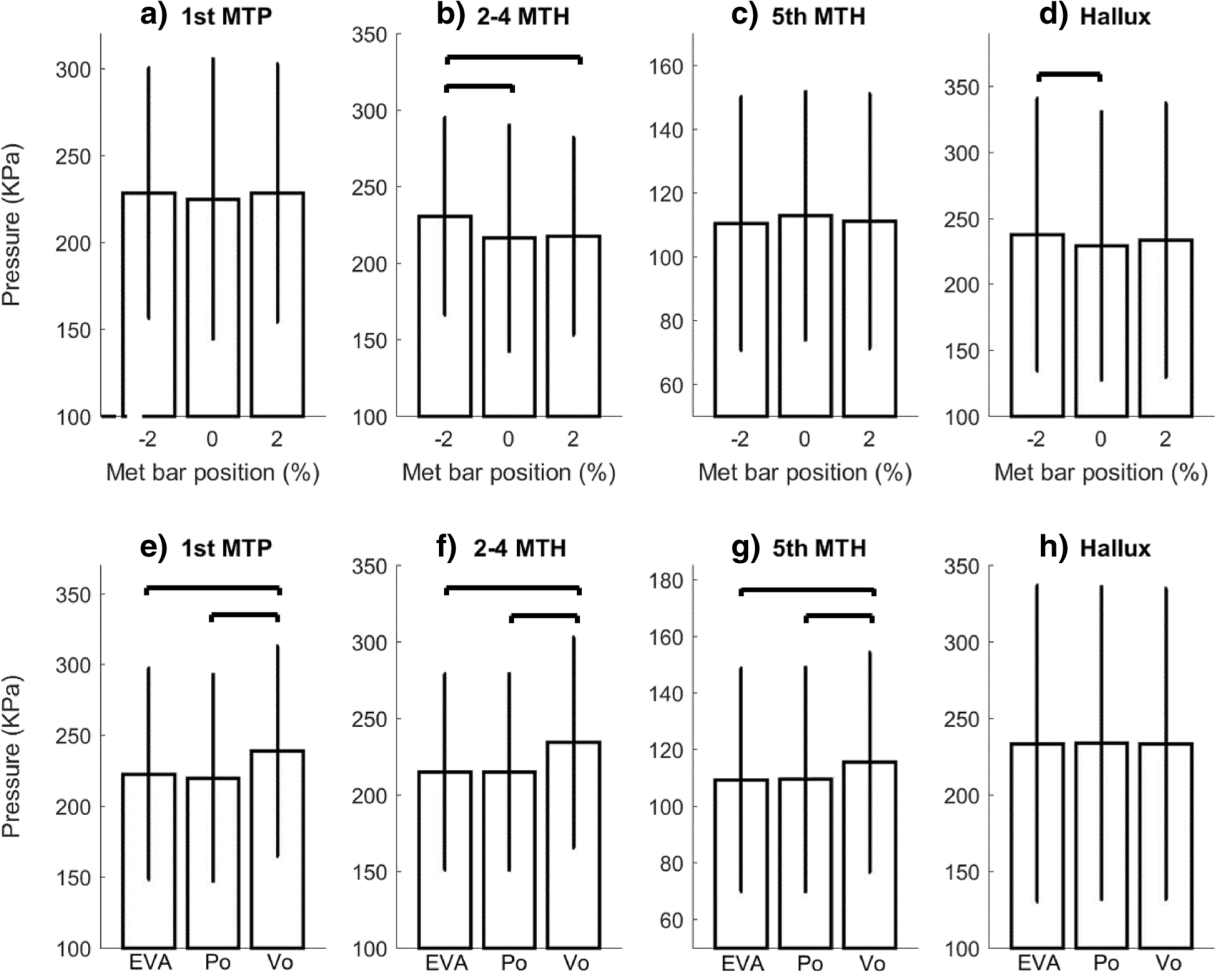
The effect of varying metatarsal bar location (**a**-**d**) and materials (**e**-**h**) on peak plantar pressures in the four different anatomical regions. Po = Poron, Vo = void. Vertical lines = standard deviation. Horizontal lines indicate significant differences (*p* < 0.05 after Bonferroni correction)

There were no differences in pressures between the 3 bar locations for MTP 1 and MTP 5. The middle and distal bar locations both statistically significantly reduced pressure compared to the proximal bar for MTP 2–4 but were not different from each other. The proximal bar location had significantly higher pressures at the hallux than the middle bar location, but not distal bar. At 1st MTP, MTP 2–4 and MTP 5, EVA and Poron statistically significantly reduced pressures compared to the void condition but were not different from each other. The hallux was not affected by changes in material choice (Fig. 3).

Based on these results a “best design” was selected from the 9 orthotic insoles, comprising the middle bar location and Poron material. In the flat insole, 61% of participants had 1 or more metatarsal head areas above the 200 KPa threshold. This was reduced to 58% by the generic “best design” orthotic insole, i.e. the middle bar location and Poron material for each participant. It was reduced to 51% when the orthotic insole design that produced the greatest pressure reduction at the site of the highest pressure was selected for each participant.

## Discussion

**Fig. 4:**
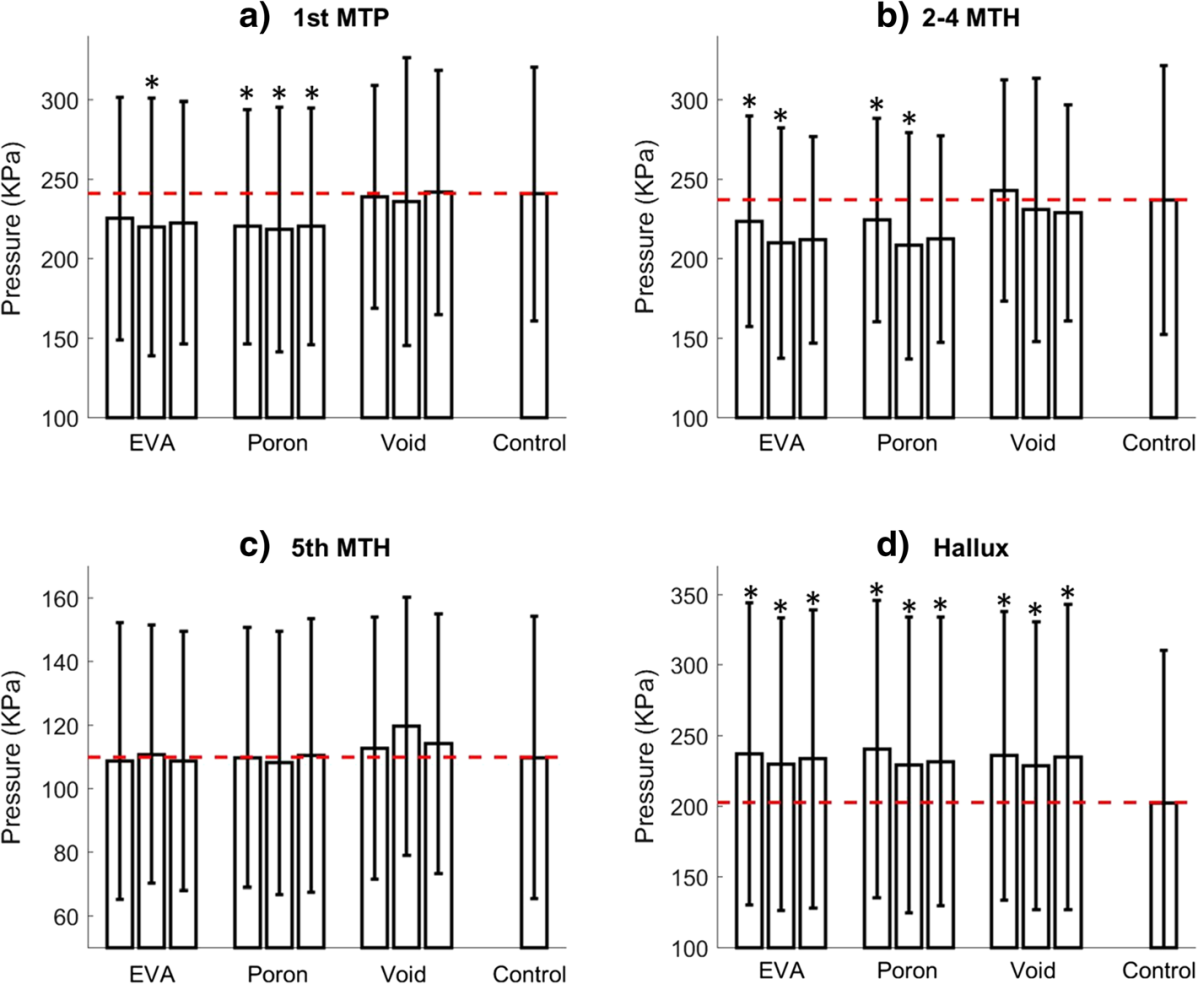
Comparison of peak pressures between the flat insole and the nine orthotic insole conditions in each of the four different anatomical regions. The three bars in void condition (EVA, Poron, Void) correspond to proximal, middle and distal metatarsal locations from left to right. The horizontal dotted line illustrates the flat insole pressure and * denotes a significant difference (*p* < 0.05) between an orthotic insole condition and the control insole following Bonferroni correction

This study focussed on pressure relieving orthotic insoles designed for retail footwear and people with diabetes and at risk of first forefoot ulceration. The aim was to investigate whether the pressure relieving effects of a customised metatarsal bar and forefoot cushioning are sensitive to bar location and material choice. By combining a metatarsal bar located at the area where plantar pressure was 77% of the peak plantar pressure, with EVA/Poron cushioning materials, it was possible to reduce pressures by up to 29 KPa under the metatarsal heads (compared to a flat control insole). Importantly, pressures were reduced to an average of 219 KPa in the 1st MTP region and 208 KPa in the 2-4th MTH region (Fig. 4). These values are only marginally above the threshold of 200 KPa suggested by Owings et al. [26] to reduce the risk of re-ulceration. This supports the belief that an appropriately customised orthotic insole could reduce pressure and we, therefore, assume the risk of first plantar ulceration.

However, selection of some aspects of orthotic design on a patient by patient basis, rather than using generic rules to locate a metatarsal bar and choose offloading material, resulted in just 7% improvement in those under feet with locations under 200 KPa (58% versus 51%). Given the extra time and cost required in producing the range of insoles required and testing them with a patient to identify the “best insole” for that patient, this is unlikely to be economically viable. It might be preferable to be able to determine, a priori, those patients would benefit most from customisation, but this would add further burden to clinical processes, taking measurements from the feet for example. Overall the question of any added value of customisation for this specific group of patients remains unanswered.

There has only been one previous study which has investigated pressure reductions from orthotic insoles contoured using both foot shape and pressure data [21]. By personalising the precise location and shape of the metatarsal bar and incorporating a void space under the metatarsal heads, Owings et al. [21] were able to achieve substantial pressure reductions, with mean peak pressures of 168 KPa (over 70 regions of interest from 40 ft). Although these peak pressures are lower than those reported in the current study, the orthotic insoles designed by Owings et al. [21] were thicker (9 mm) and could only be accommodated within extra depth shoes. The contrast between studies illustrates the trade-off between orthotic insole thickness and the magnitude of pressure reduction when footwear choice limits insole design. We suggest that because our insoles are thinner and accommodated in retail footwear, they are more likely to be worn by people who consider themselves at low risk of ulceration (because they have no prior experience of ulceration).

This is the first study to investigate the effect of systematically varying metatarsal bar location relative to plantar pressure distribution. Based on our data, we suggest that the anterior border of the metatarsal bar should be located at the point at which pressure reaches approximately 70–77% of the peak value (between proximal and middle bar position in this study). We used a 3D foot scanner and plantar pressure measurement plate to inform the precise location and shape of the metatarsal bar and offloading material. However, these are available to few practitioners, and further work is required to understand whether an appropriate level of precision could be achieved using simpler, less expensive approaches.

Previous studies which have investigated the effect of metatarsal bar/pad location have typically located the bar/pad relative to the metatarsal heads [36]. Interestingly, contrasting recommendations have been provided, one study suggesting the metatarsal bar should be located 6-11 mm proximal to the metatarsal heads [22] and another 5 mm distal [24]. It is possible that different results reflect the differing populations investigated, with Hastings et al. [22] studying a group with diabetes and Lee et al. [24] a healthy older group. However, it is also possible that this is a consequence of variation in anatomical structures. Similar to the approach proposed by Hsi et al. [37], we located the metatarsal bar proximal to the location of peak pressure, somewhat independent of structural information, and were able to achieve mean peak pressures just above the re-ulceration threshold of 200 KPa [26]. We, therefore, propose that optimal clinical results could be achieved if metatarsal bars/pad are located according to regions of peak pressure, rather than anatomical structures.

The orthotic insoles tested in this study incorporated a small void/space directly distal to the metatarsal bar. When this void/space was occupied by cushioning materials (EVA or Poron), pressures decreased regardless of metatarsal bar location. Interestingly, although previous research has demonstrated reductions in plantar pressures using orthotic insoles which are made completely of cushioning materials [38], there has been minimal research into orthotic insoles which combine materials of different densities. Our findings are consistent with data from a finite element model [18], which demonstrated reductions in plantar pressure when orthotic insoles incorporate softer material under the metatarsal heads. Thus, in contrast to the approach of Owings et al. [21], in which only a void/space was incorporated into the orthotic insole, we suggest that optimal pressure results will be obtained if cushioning materials are located under the metatarsal heads.

Although our data demonstrated reductions in pressure under the metatarsal heads, peak pressures were elevated under the hallux (Fig. 4d). This is consistent with observations from studies of cushioning materials [18]. Peak pressures on the hallux typically occur in the later stages of stance when the load is distributed primarily across the forefoot and toes. During this phase, the raised profile of the metatarsal bar, and corresponding increased height of the metatarsal structures [39] would appear to shift load onto the hallux. Given the magnitude of increases in pressure in this region (Fig. 4), clinicians need to decide whether the hallux is a greater priority for pressure relief than metatarsal heads. Alternative insole designs or pressure-reducing footwear [40] may be more appropriate in these cases.

There are two primary of limitations to this study which need to be acknowledged. Firstly, our focus was on plantar pressure and we did not prospectively measure ulceration rates, and we, therefore, cannot conclude that ulceration rate would be reduced by the insole designs. The aetiology of ulceration is complex and factors other than plantar pressure play a role. Another limitation is that, although most participants were able to maintain a walking speed with 5% of the target (self-selected speed), for some participants we were forced to relax this limit to 10% between different orthotic insole conditions. This inability to walk within a tightly controlled speed is likely a consequence of neuropathy which has been associated with increased gait variability [41]. It is possible that these differences in speed could have increased the variability in plantar pressure measurements. However, we used a within-subjects design and most participants walked within 5%, we ranked insoles by efficacy (which may be less sensitive to variations due to variation in speed), our analysis focuses on the mean across *n* = 60 participants. We, therefore, believe speed-related variability is likely to have had minimal effect on our final conclusions.

## Conclusions

In summary, we used foot shape and plantar pressure data to produce a customised orthotic insole design to offload the forefoot in people with diabetes whilst they walk in retail footwear. Mean peak plantar pressures in optimal orthotic insole design were only marginally above the critical threshold of 200 KPa suggested by Owings et al. [26].

## Data Availability

All data generated or analysed during this study are included in this published article.

## Abbreviations

MTH: Metatarsal heads
MTP: Metatarsophalangeal
